# Primary central nervous system lymphoma: is absence of intratumoral hemorrhage a characteristic finding on MRI?

**DOI:** 10.1515/raon-2015-0007

**Published:** 2015-03-25

**Authors:** Akihiko Sakata, Tomohisa Okada, Akira Yamamoto, Mitsunori Kanagaki, Yasutaka Fushimi, Toshiki Dodo, Yoshiki Arakawa, Jun C Takahashi, Susumu Miyamoto, Kaori Togashi

**Affiliations:** 1 Department of Diagnostic Imaging and Nuclear Medicine, Kyoto University Graduate School of Medicine, Kyoto, Japan; 2 Department of Neurosurgery, Kyoto University Graduate School of Medicine, Kyoto, Japan

**Keywords:** glioblastoma multiforme, primary central nervous system lymphoma, magnetic resonance imaging

## Abstract

**Background.:**

Previous studies have shown that intratumoral hemorrhage is a common finding in glioblastoma multi-forme, but is rarely observed in primary central nervous system lymphoma. Our aim was to reevaluate whether intratumoral hemorrhage observed on T2-weighted imaging (T2WI) as gross intratumoral hemorrhage and on susceptibility-weighted imaging as intratumoral susceptibility signal can differentiate primary central nervous system lymphoma from glioblastoma multiforme.

**Patients and methods.:**

A retrospective cohort of brain tumors from August 2008 to March 2013 was searched, and 58 patients (19 with primary central nervous system lymphoma, 39 with glioblastoma multiforme) satisfied the inclusion criteria. Absence of gross intratumoral hemorrhage was examined on T2WI, and an intratumoral susceptibility signal was graded using a 3-point scale on susceptibility-weighted imaging. Results were compared between primary central nervous system lymphoma and glioblastoma multiforme, and values of *P* < 0.05 were considered significant.

**Results.:**

Gross intratumoral hemorrhage on T2WI was absent in 15 patients (79%) with primary central nervous system lymphoma and 23 patients (59%) with glioblastoma multiforme. Absence of gross intratumoral hemorrhage could not differentiate between the two disorders (*P* = 0.20). However, intratumoral susceptibility signal grade 1 or 2 was diagnostic of primary central nervous system lymphoma with 78.9% sensitivity and 66.7% specificity (*P* < 0.001), irrespective of gross intratumoral hemorrhage.

**Conclusions.:**

Low intratumoral susceptibility signal grades can differentiate primary central nervous system lymphoma from glioblastoma multiforme. However, specificity in this study was relatively low, and primary central nervous system lymphoma cannot be excluded based solely on the presence of an intratumoral susceptibility signal.

## Introduction

Primary central nervous system lymphoma (PCNSL) represents approximately 2–6% of all brain tumors and 1–2% of all non-Hodgkin lymphomas.^1^,^2^ The vast majority of PCNSLs are diffuse large B-cell lymphomas, regardless of the patient’s immunological state, and primary T-cell lymphomas are uncommon in the central nervous system (CNS).^3^ The incidence of this pathology has recently been on the rise among both immunocompetent and immunocompromised populations.^4^

Histology is required for a definitive diagnosis, but radical surgical excision of PCNSL is not warranted; even partial tumor resection seems to be a negative prognostic factor.^2^ Glioblastoma multiforme (GBM), on the other hand, requires maximal excision. Because symptomatic patients with PCNSL often present with lesions of considerable size, steroid administration is sometime clinically indicated before pathological confirmation, resulting in low diagnostic yields for histological examination.^5^ Accurate diagnosis of PCNSL by initial imaging is thus crucial to avoid steroid treatment and facilitate biopsy, rather than resection that does not improve prognosis.

PCNSL has many documented imaging features^1^,^6^–^10^, but may mimic other diseases or show atypical findings, making preoperative diagnosis of PCNSL imperfect even with current advanced imaging techniques.^11^,^12^ As another imaging method, susceptibility-weighted imaging (SWI) has recently been reported to differentiate PCNSL from GBM, as the most common malignant primary brain tumor, nearly perfectly when intratumoral susceptibility signal (ITSS) that reflects hemorrhage is used.^13^–^15^

However, some reports have described PCNSL with cerebral hemorrhage even in non-HIV patients^16^–^18^ and hemorrhage may not be particularly rare even among immunocompetent patients. Moreover, the relationship between ITSS and intratumoral hemorrhage observed on conventional imaging modalities such as computed tomography (CT) or T2-weighted imaging (T2WI) in PCNSL cases has not been clarified in detail.^13^–^15^

Given these considerations, we have retrospectively reviewed all patients with PCNSL and GBM encountered within a fixed period in our hospital to investigate the differential capabilities of gross intratumoral hemorrhage (GITH) on T2WI and ITSS grading on SWI.

## Patients and methods

### Patients

A retrospective cohort of brain tumors in the pathology archives of our institution from August 2008 to March 2013 was searched for cases of PCNSL and GBM. From the database, we included all patients with pathologically diagnosed PCNSL and GBM who had preoperative CT and MRI studies including T2WI and SWI. From the database, we found 65 patients with pathologically diagnosed PCNSL and GBM who had undergone preoperative CT and MRI studies, including T2WI and SWI. Exclusion criteria were a history of organ transplantation (n = 1) or surgical intervention (*i.e.* biopsy or drainage) before initial MRI (n = 6). In total, 58 patients satisfied these criteria: 19 patients with PCNSL (10 men, 9 women; mean age, 65.1 years; range, 26–83 years) and 39 patients with GBM (24 men, 15 women; mean age, 59.7 years; age range, 16–87 years). Among PCNSL patients, all were diagnosed with B-cell lymphoma (diffuse large B-cell lymphoma, n = 17; precursor B cell lymphoma, n = 1), except 1 case of T-cell lymphoma. No patient had history of acquired immunodeficiency syndrome or other immunodeficiency disorders. One patient with B-cell lymphoma had Sjögren syndrome and was treated with disease-modifying anti-rheumatic drugs. Another patient was treated for multiple sclerosis with steroid pulse therapy for 1 month prior to admission to our hospital. One patient with T-cell lymphoma showed positive results for human T-lymphotropic virus type 1 infection postoperatively, but the lesion was limited to the CNS. Five patients with PCNSL had received steroids prior to MRI to diminish edema. This study was approved by the institutional review board. Given the retrospective design, the requirement for informed consent was waived.

### Image acquisition

Patients were imaged using a 3T MRI system (Magnetom Trio Tim or Skyra; Siemens Healthcare, Erlangen, Germany) with a 32-channel head coil. T2WI was acquired using a fast spin-echo sequence under the following conditions: repetition time (TR), 3200 ms; echo time (TE), 79 ms; matrix, 420 × 448; field of view, 206×220 mm; matrix size, 0.49 × 0.49 mm; 35 slices of 3 mm thickness with a 1 mm gap. SWI was acquired with a three-dimensional fully flow-compensated gradient echo sequence using the following parameters: TR, 28 ms; TE, 20 ms; flip angle, 15°; matrix, 320 × 230; field of view, 230 × 179 mm; matrix size, 0.72 × 0.78 mm. Slab size was 76.8 mm or 128 mm, partitioned into 64 slices of 1.2 or 2 mm (n = 28 and 30, respectively). SWI sequences were acquired before contrast administration. Diffusion-weighted imaging (DWI), pre- and post-contrast enhanced T1-weighted images were also routinely acquired. DWI was performed using a single-shot spin-echo (SE) echo planar sequence with following parameters: TR 5000ms, TE 84 ms, flip angle 90°, matrix 160 × 160, field of view 220 × 220 mm, matrix size, 0.49 × 0.49 mm; 35 slices of 3 mm thickness with a 1 mm gap. Diffusion-sensitizing gradients were applied in 3 directions with b factors of 0 and 1000 s/mm^2^. Apparent diffusion coefficients (ADCs) were automatically calculated by the operating console of the MR scanner and displayed as corresponding ADC maps. All patients underwent unenhanced CT with an in-plane resolution of 0.41 × 0.41 mm and slice thicknesses of 4–8 mm in conventional scans and 5 mm in helical acquisitions using 16- or 64-detector-row CT scanners (Aquilion 16 or Aquilion 64; Toshiba Medical Systems, Ohtawara, Japan).

### Image analysis

**FIGURE 1.**
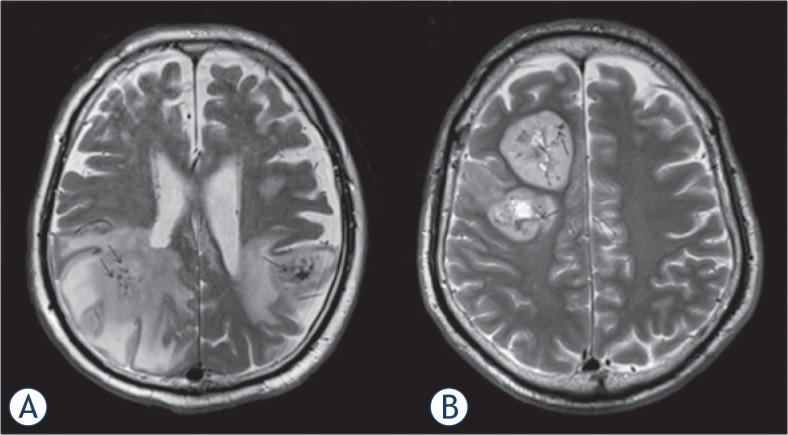
Gross intratumoral hemorrhage (arrows) in primary central nervous system lymphoma **(A)** and glioblastoma multiforme **(B)** on T2-weighted image. Both cases show low-intensity areas representing intratumoral hemorrhage.

**FIGURE 2.**
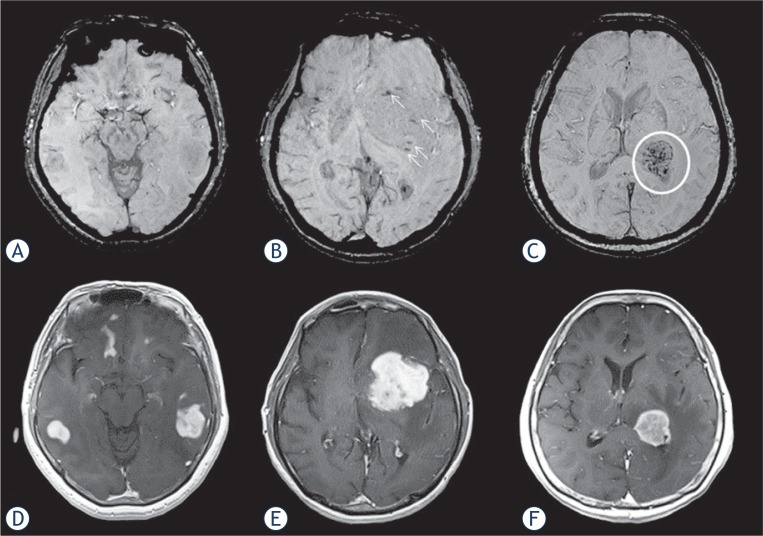
Intratumoral susceptibility signals in patients with primary central nervous system lymphoma: **(A)** Grade 1: multifocal tumors in bilateral temporal lobes show no intratumoral susceptibility signal on susceptibility-weighted imaging (SWI). **(B)** Grade 2: tumor in the left basal ganglia shows punctate low-intensity signals (arrows) on SWI. **(C)** Grade 3: tumor in the left thalamus shows multiple linear or nodular low-intensity signals (circle) on SWI. **(D–F)** Contrast-enhanced T1-weighted imaging shows primary central nervous system lymphomas with intense enhancement.

Qualitative analysis including T2WI and contrast-enhanced T1-weighted images (CE-T1WI) as well as SWI was conducted on a clinical picture archiving and communication system (Centricity, PACS workstation version 3.2; GE Medical Systems, Milwaukee, WI) by two board-certified neuroradiologists (A.S. and T.D.; both with 5 years of experience in diagnostic radiology) who were blinded to the final diagnosis. First, they evaluated the absence of GITH. GITH was defined as nodular or linear hypo-intense foci observed on T2WI (Figure 1). Second, ITSS grading on SWI was conducted independently by the same two neuroradiologists at 1 month after GITH evaluation. As described by Kim *et al.*^12^, ITSS was defined as a dot-like or fine linear low signal within a tumor, and graded using a 3-point scale: grade 1, no ITSS (Figure 2A); grade 2, 1–10 ITSSs (Figure 2B); and grade 3, ≥ 11 ITSSs (Figure 2C) within a tumor. Corresponding contrast-enhanced T1-weighted images are presented as tumor references (Figure 2D–F). Enhancement patterns on contrast-enhanced T1-weighted image were evaluated as necrotic or non-necrotic: necrotic was defined as solid enhancement with any loss of contrast enhancement.^19^ When absence of GITH, ITSS grading or enhancement patterns were discordant between evaluators, the final results were reached by consensus. To exclude low signal intensity caused by calcification (defined as a CT attenuation value >100 Hounsfield units), unenhanced CT was also referred to.

The same neuroradiologists (A.S. and T.D.) evaluated ADC maps using software (Image J version 1.49a; NIH, Bethesda). Firstly, we selected all slices that included tumor. One round- or oval-shaped region of interest (area, approximately 0.3 cm^2^) was carefully placed on each slice of the ADC map of the whole tumor to include the area with the lowest ADC value determined by visual inspection. Cystic, necrotic or hemorrhagic areas were carefully avoided by referencing to conventional MR images. Finally, the value of a region of interest with the lowest average ADC value was selected as a minimum ADC (ADCmin) value of the tumor in each case.^20^

### Statistical analysis

Patient groups of PCNSL and GBM were compared for age, sex and parameters using a *t*-test and a Pearson’s χ^2^ test, respectively. Inter-observer variability of GITH and ITSS grading was evaluated using κ statistics. Inter-observer variability of the readers for ADC analysis was evaluated by intraclass correlation (ICC) coefficient (0.00–0.20 poor, 0.21–0.40 fair, 0.41–0.60 moderate, 0.61–0.80 good and 0.81–1.00 excellent correlation). ADCs were averaged between the two observers for further analysis. ADCmin Receiver-operating characteristic (ROC) curve analysis was conducted for GITH, ITSS, enhancement pattern and ADCmin, and sensitivity, specificity, positive predictive value (PPV) and negative predictive value (NPV) were calculated for differentiating PCNSL from GBM.^21^ Areas under the curve (AUCs) were statistically compared using a method by Delong *et al*.^22^ To analyze whether ADCmin were affected by microhemorrhage, ADCmin values of the groups defined by ITSS were compared using ANOVA in both GBM and PCNSL patients. A value of *P* < 0.05 was considered significant. All statistical analyses were conducted using MedCalc for Windows (version 12.5.0.0; MedCalc Software, Mariakerke, Belgium).

## Results

No significant differences in age (*P* = 0.39) or sex (*P* = 0.72) were found between patient groups. Inter-observer agreement between the two evaluators was substantial for GITH (κ = 0.75, 95% confidence interval (CI) = 0.58–0.92) and almost perfect for ITSS (κ = 0.88, 95% CI = 0.81–0.96). ICC of ADCmin was 0.693 (95% CI = 0.48–0.82).

**FIGURE 3.**
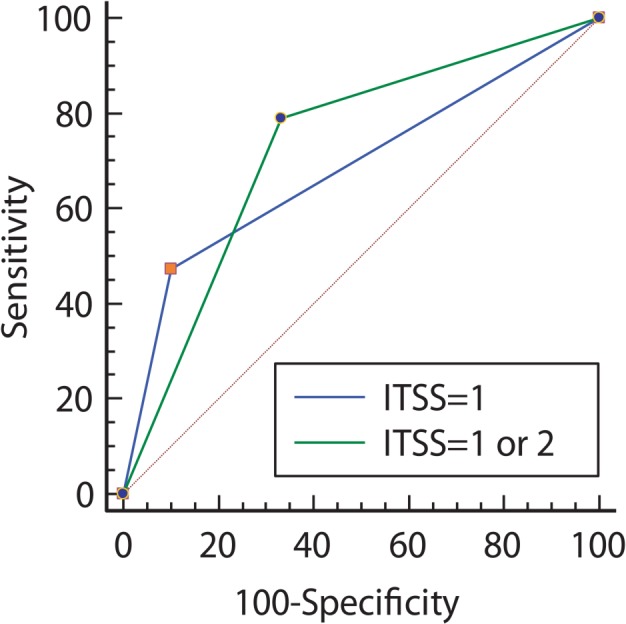
Receiver-operating characteristic curve analysis of intratumoral susceptibility signal (ITSS) grading to differentiate primary central nervous system lymphoma from glioblastoma multiforme. ITSS grades≤ 2 is diagnostic of primary central nervous system lymphoma with 78.9% sensitivity and 66.7% specificity, not as high as in previous studies.

CT showed no calcification in any cases. GITH was not observed in 15 patients (79%) with PCNSL and in 23 patients (59%) with GBM (Table 1), and ROC analysis thus failed to reveal any significant difference (*P* = 0.20). ITSS grades 1, 2 and 3 were found in 9, 6 and 4 PCNSL patients, respectively, and in 4, 9 and 26 GBM patients, respectively (Table 2). GITHs were associated with grade 3 ITSS in almost all cases, except for 1 patient with GBM and 1 patient with PCNSL; both of these patients showed grade 2 ITSS. ITSS was not observed (*i.e*., grade 1) in 9 PCNSL patients (47.4%) and 4 GBM patients (10.3%). When ITSS grade 1 was used as the criterion of PCNSL, sensitivity, specificity, PPV, and NPV was 47.4%, 89.7%, 69.2%, and 77.8% respectively, whereas ITSS grade 1 or 2 was diagnostic of PCNSL with 78.9%, 66.7%, 53.6% and 86.7%. The AUC values were 0.686 and 0.728, respectively (*p* values were 0.0036 and 0.0002, Figure 3). There was not statistically significant difference between the two curves.

Enhancement patterns of GBM and PCNSL were summarized in Table 3: one patient with no contrast-enhancement was excluded from this analysis. Most GBMs showed necrotic pattern, while most PCNSL showed non-necrotic enhancement. Sensitivity, specificity PPV and NPV were 83.3%, 94.9%, 88.2% and 92.5%, respectively.

**FIGURE 4.**
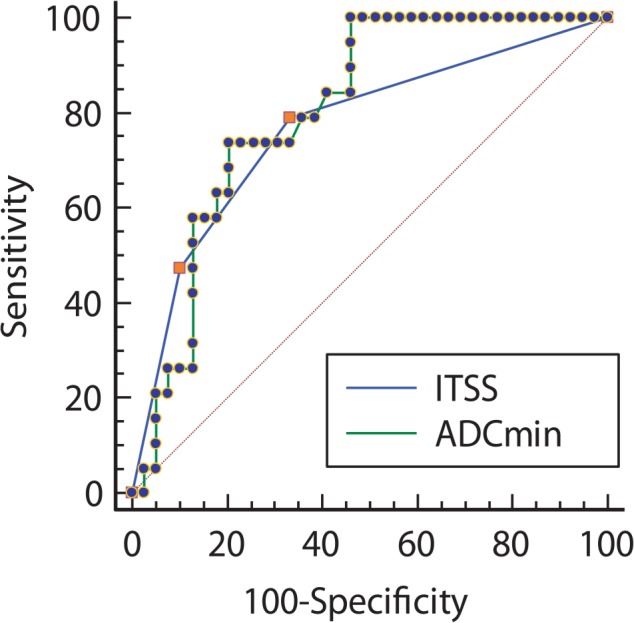
Receiver-operating characteristic curve analysis of minimum apparent diffusion coefficient (ADCmin) and intratumoral susceptibility signal (ITSS) grading to differentiate primary central nervous system lymphoma from glioblastoma multiforme. ADCmin ≤ 0.629 mm^2^/s is diagnostic of primary central nervous system lymphoma with 100% sensitivity and 53.8% specificity.

ADCmin was significantly lower for lymphoma than for GBM (0.487 ± 0.09 × 10^−3^ mm^2^/s and 0.645 ± 0.15 × 10^−3^ mm^2^/s, respectively; *p* = 0.001 with student *t*-test). ROC curve analysis of ADCmin found the optimal cutoff value to be 0.629 mm^2^/s (sensitivity: 100%, specificity: 53.8%, PPV: 51.4%. and NPV: 100%). There was not statistically significant differences between AUCs of ITSS and ADCmin (*p* value = 0.68) (Figure 4).

## Discussion

Our results demonstrated that ITSS on SWI can aid to differentiate PCNSL from GBM compared with GITH on T2WI. However, in this study cohort, more than half of PCNSL patients showed positive ITSS (*i.e*., ≥ 2), including 4 patients with ITSS grade 3. The frequency of positive ITSS in PCNSL was much higher than in previous reports, resulting in the lower specificity of 66.7%.

Conventional imaging technique, especially CE-T1WI, is useful for differentiating PCNSL and GBM. Most GBMs show heterogeneous enhancement with variable size of necrotic foci, while PCNSL typically shows homogenous enhancement because of paucity of intratumoral necrosis, especially in immunocompetent patients. However, up to 10% of patients show atypical enhancement patterns in GBM and PCNSL.^1^,^19^ Therefore, further imaging technique is warranted for more appropriate evaluation.

Hemorrhagic and necrotic foci are commonly observed in GBM.^23^ Previous studies have also showed that ITSS on T2*-weighted imaging or SWI is more frequently detected in high-grade glioma.^24^,^25^ Conversely, in immunocompetent PCNSL patients, intratumoral hemorrhage before therapy has rarely been observed. On CT and conventional MRI, tumor-associated hemorrhage was reported in 0–8% of PCNSL cases.^1^,^6^,^8^,^10^ Based on this rarity, the presence of hemorrhage or calcification is used to exclude PCNSL from the differential diagnosis.

Previous reports on SWI have confirmed non-SWI results.^13^–^15^,^21^,^26^ Kim *et al.,* first demonstrated the presence of ITSS, particularly grade 3, could distinguish high-grade glioma from PCNSL with 100% specificity, although their study included only 7 patients with PCNSL.^13^ Radbruch *et al*., also recently reported that ITSS was not observed in any of 14 patients with B-cell PCNSL, with the exception of 1 case of T-cell lymphoma.^14^ Peters *et al.,* also showed that SWI is useful for differentiating PCNSL from GBM, although 1 in 4 patients with PCNSL showed grade 2 ITSS.^15^ Previous studies have suggested nearly complete absence of ITSS in PCNSL.^13^–^15^,^21^,^26^

However, on pathological examination, necrosis and hemorrhage are occasionally observed in PCNSL.^27^ Some case reports have described PCNSL presenting with intracerebral hemorrhage in both immunocompetent and immunocompromised patients.^16^–^18^ In a study of 10 immunocompetent PCNSL patients, small hemorrhage was found on MRI in 4 of 19 lesions (21%).^9^ Moreover, Kickingereder *et al.*, have recently reported presence of the ITSS in 6 out of 19 PCNSL patients on 3T MRI.^19^ These findings are comparable to our result. SWI is much more sensitive to susceptibility difference than conventional MRI, including T2*-weighted imaging^24^,^28^,^29^, and is considered to identify more small hemorrhagic lesions within the tumors. Actually, in the present study, patients with positive ITSS were twice as common as those with GITH in both groups. This is in accordance with the recent report by Ding *et al.*, demonstrating the significant difference in the detecting rate of intra-tumoral hemorrhage in patients with GBM and brain metastasis between the conventional MR imaging and SWI.^21^ Therefore, conventional MR including T2^*^WI seems insensitive to hemorrhagic change, and unsuitable for differentiating GBM from PCNSL based on the absence of intratumoral hemorrhage.

This study showed much higher frequency of positive ITSS in PCNSL patients compared with previous studies. Several factors may contribute to this result. One possible explanation is that SWI in this study used thinner slices than previous studies. Nandigum *et al.,* demonstrated that SWI acquired with thinner slices in a higher magnetic field detected significantly more cerebral microbleeds.^30^ Previous SWI studies on PCNSL have reported almost no ITSS^13^,^14^, but used 2.5- or 3-mm slice thicknesses, thicker than used in this study (*i.e*., 1.2 or 2 mm). Peters *et al*., used 1-mm slice thickness and found grade 2 ITSS in 1 of 4 patients with PCNSL (diffuse large B-cell lymphoma).^15^ Thinner slice thickness thus appears more sensitive to small foci of hemorrhage and may have contributed to higher ITSS grades in our PCNSL cases compared to previous reports. The other technical factor is the difference in head coils used in studies. Previous studies used 8- to 12-channel head coils^13^–^15^, whereas the present study used a 32-channel brain coil, which improves the signal-to-noise ratio (SNR).^31^ SWI was acquired at high resolution, but there is a trade-off between resolution and SNR. With lower SNR, lesion detectability is impaired^32^,^33^, which may also have contributed to the differences with previous studies.

Several authors investigate the role of other advanced techniques, especially DWI, in evaluation of differentiation of PCNSL and GBM.^12^,^34^,^35^ Generally, the ADC values of PCNSL are low, reflecting the high degree of cellularity. However, according to several earlier investigations, the differences in ADC between patients with PCNSL and those with glioblastoma were not always statistically significant.^35^ Our results showed there were significant differences between both groups, but its specificity was relatively low, which is consistent with previous studies. We also tested if the presence of ITSS effects ADC values, but the ADC value of the tumor did not differ depending on the amount of microhemorrhage.

Our results showed the diagnostic capability of ITSS for differentiating PCNSL from GBM were comparable to that of ADCmin, however, as a single parameter, both of them were not so specific as previously described.^13^–^15^,^21^,^26^ Kickingereder *et al.,* showed that ITSS as an additional imaging parameter allowed correct classification of the atypical GBM (*i.e*. GBM showing homogenous enhancement) and PCNSL.^19^ Therefore, considering with the limited diagnostic capability of ITSS, combined analysis of several parameters obtained by other imaging technique, such as PWI^12^, ASL^26^ and MRS^19^ should be considered in the clinical practice.

Some limitations in this study must be considered. The sample size was relatively small. However, the case number is more than or comparable to former studies. Another limitation was that we included patients who were treated with steroids before imaging. Steroid therapy is well known to disrupt cellular morphology, which may affect imaging appearance.^5^,^36^ However, no patients with steroid administration prior to MRI showed positive ITSS, and steroid use had minimal effect on our results. We also included one human T-lymphotropic virus type 1-positive case that may potentially have arisen in an immunocompromised patient. PCNSL in an immunocompromised host with primary CNS T-cell lymphoma is known to have a relatively high incidence of intratumoral hemorrhage^11^,^37^, but the patient in this study showed no GITH or ITSS. Finally, Epstein-Barr virus infection is another important factor that must be considered. Lee *et al.,* recently demonstrated that Epstein-Barr virus -positive PCNSL cases showed intratumoral necrosis or hemorrhage more frequently than cases with Epstein-Barr virus -negative PCNSL, even in the absence of HIV infection.^38^ No description of Epstein-Barr virus infection status has been available in previous studies on ITSS^13^–^15^, and this was also the case for this study. PCNSL with GITH or ITSS could potentially be related to Epstein-Barr virus infection, but no conclusion can be drawn within this study.

In conclusion, low ITSS grades can differentiate PCNSL from GBM. However, specificity in this study was relatively low, and PCNSL cannot be excluded based solely on the presence of an ITSS. Careful evaluation using several imaging technique is important.

## Figures and Tables

**TABLE 1.** Gross intratumoral hemorrhage (GITH) frequency in primary central nervous system lymphoma (PCNSL) and glioblastoma multiforme (GBM)

**Pathological Diagnosis**	**GITH**	

	**Negative (%)**	**Positive (%)**
PCNSL	15 (79)	4 (21)
GBM	23 (59)	16 (41)

**TABLE 2.** Intratumoral susceptibility signal (ITSS) grading of primary central nervous system lymphoma (PCNSL) and glioblastoma multiforme (GBM)

**Pathological Diagnosis**	**ITSS grading**		

	**Grade 1 (%)**	**Grade 2 (%)**	**Grade 3 (%)**
PCNSL	9 (47)	6 (32)	4 (21)
GBM	4 (10)	9 (23)	26 (67)

**TABLE 3.** Enhancement patterns of primary central nervous system lymphoma (PCNSL) and glioblastoma multiforme (GBM)

**Enhancement pattern**	**Pathological Diagnosis**	

	**PCNSL**	**GBM**
Non-necrotic	15	2
Necrotic	3	37

## References

[1] Haldorsen IS, Kråkenes J, Krossnes BK, Mella O, Espeland A (2009). CT and MR imaging features of primary central nervous system lymphoma in Norway, 1989–2003. AJNR Am J Neuroradiol.

[2] Bataille B, Delwail V, Menet E, Vandermarcq P, Ingrand P, Wager M (2000). Primary intracerebral malignant lymphoma: report of 248 cases. J Neurosurg.

[3] Schlegel U, Schmidt-Wolf IG, Deckert M (2000). Primary CNS lymphoma: clinical presentation, pathological classification, molecular pathogenesis and treatment. J Neurol Sci.

[4] Olson JE, Janney CA, Rao RD, Cerhan JR, Kurtin PJ, Schiff D (2002). The continuing increase in the incidence of primary central nervous system non-Hodgkin lymphoma: a surveillance, epidemiology, and end results analysis. Cancer.

[5] Geppert M, Ostertag CB, Seitz G, Kiessling M (1990). Glucocorticoid therapy obscures the diagnosis of cerebral lymphoma. Acta Neuropathol.

[6] Zhang D, Hu L-B, Henning TD, Ravarani EM, Zou LG, Feng XY (2010). MRI findings of primary CNS lymphoma in 26 immunocompetent patients. Korean J Radiol.

[7] Bühring U, Herrlinger U, Krings T, Thiex R, Weller M, Küker W (2001). MRI features of primary central nervous system lymphomas at presentation. Neurology.

[8] Jenkins CN, Colquhoun IR (1998). Characterization of primary intracranial lymphoma by computed tomography: an analysis of 36 cases and a review of the literature with particular reference to calcification haemorrhage and cyst formation. Clin Radiol.

[9] Ueda F, Takashima T, Suzuki M, Kadoya M, Yamashita J, Kida T (1995). MR imaging of primary intracranial malignant lymphoma. Radiat Med.

[10] Coulon A, Lafitte F, Hoang-Xuan K, Martin-Duverneuil N, Mokhtari K, Blustajn J (2002). Radiographic findings in 37 cases of primary CNS lymphoma in immunocompetent patients. Eur Radiol.

[11] Haldorsen IS, Espeland A, Larsson E-M (2011). Central nervous system lymphoma: characteristic findings on traditional and advanced imaging. AJNR Am J Neuroradiol.

[12] Yamashita K, Yoshiura T, Hiwatashi A, Togao O, Yoshimoto K, Suzuki SO (2013). Differentiating primary CNS lymphoma from glioblastoma multiforme: assessment using arterial spin labeling, diffusion-weighted imaging, and ą^8^F-fluorodeoxyglucose positron emission tomography. Neuroradiology.

[13] Kim HS, Jahng GH, Ryu CW, Kim SY (2009). Added value and diagnostic performance of intratumoral susceptibility signals in the differential diagnosis of solitary enhancing brain lesions: preliminary study. AJNR Am J Neuroradiol.

[14] Radbruch A, Wiestler B, Kramp L, Lutz K, Bäumer P, Weiler M (2013). Differentiation of glioblastoma and primary CNS lymphomas using susceptibility weighted imaging. Eur J Radiol.

[15] Peters S, Knöß N, Wodarg F, Cnyrim C, Jansen O (2012). Glioblastomas vs. lymphomas: more diagnostic certainty by using susceptibility-weighted imaging (SWI). Rofo.

[16] Rubenstein J, Fischbein N, Aldape K, Burton E, Shuman M (2002). Hemorrhage and VEGF expression in a case of primary CNS lymphoma. J Neurooncol.

[17] Kim IY, Jung S, Jung TY, Kang SS, Choi C (2008). Primary central nervous system lymphoma presenting as an acute massive intracerebral hemorrhage: case report with immunohistochemical study. Surg Neurol.

[18] Kimura N, Ishibashi M, Masuda T, Ito M, Takahashi Y, Kumamoto T (2009). Primary central nervous system lymphoma with cortical laminar hemorrhage. J Neurol Sci.

[19] Kickingereder P, Wiestler B, Sahm F, Heiland S, Roethke M, Schlemmer HP (2014). Primary central nervous system lymphoma and atypical glioblastoma: multiparametric differentiation by using diffusion-, perfusion-, and susceptibility-weighted MR imaging. Radiology.

[20] Higano S, Yun X, Kumabe T, Watanabe M, Mugikura S, Umetsu A (2006). Malignant astrocytic tumors: clinical importance of apparent diffusion coefficient in prediction of grade and prognosis. Radiology.

[21] Ding Y, Xing Z, Liu B, Lin X, Cao D (2014). Differentiation of primary central nervous system lymphoma from high-grade glioma and brain metastases using susceptibility-weighted imaging. Brain and Behavior.

[22] DeLong ER, DeLong DM, Clarke-Pearson DL (1988). Comparing the areas under two or more correlated receiver operating characteristic curves: a nonparametric approach. Biometrics.

[23] Kondziolka D, Bernstein M, Resch L, Tator CH, Fleming JF, Vanderlinden RG (1987). Significance of hemorrhage into brain tumors: clinicopathological study. J Neurosurg.

[24] Bagley LJ, Grossman RI, Judy KD, Curtis M, Loevner LA, Polansky M (1997). Gliomas: correlation of magnetic susceptibility artifact with histologic grade. Radiology.

[25] Li C, Ai B, Li Y, Qi H, Wu L (2010). Susceptibility-weighted imaging in grading brain astrocytomas. Eur J Radiol.

[26] Furtner J, Schöpf V, Preusser M, Asenbaum U, Woitek R, Wöhrer A (2014). Non-invasive assessment of intratumoral vascularity using arterial spin labeling: A comparison to susceptibility-weighted imaging for the differentiation of primary cerebral lymphoma and glioblastoma. Eur J Radiol.

[27] Deckert M, Paulus W, Louis DN, Ohgaki H, Wiestler OD, Cavenee WK (2007). Malignant lymphoma. WHO classification of tumours of the central nervous system.

[28] Haacke EM, Mittal S, Wu Z, Neelavalli J, Cheng YC (2009). Susceptibility-weighted imaging: technical aspects and clinical applications, part 1. AJNR Am J Neuroradiol.

[29] Mittal S, Wu Z, Neelavalli J, Haacke EM (2009). Susceptibility-weighted imaging: technical aspects and clinical applications, part 2. AJNR Am J Neuroradiol.

[30] Nandigam RN, Viswanathan A, Delgado P, Skehan ME, Smith EE, Rosand J (2009). MR imaging detection of cerebral microbleeds: effect of susceptibility-weighted imaging, section thickness, and field strength. AJNR Am J Neuroradiol.

[31] Wiggins GC, Triantafyllou C, Potthast A, Reykowski A, Nittka M, Wald LL (2006). 32-channel 3 Tesla receive-only phased-array head coil with soccer-ball element geometry. Magn Reson Med.

[32] Constable RT, Henkelman RM (1991). Contrast, resolution, and detectability in MR imaging. J Comput Assist Tomogr.

[33] Kale SC, Chen XJ, Henkelman RM (2009). Trading off SNR and resolution in MR images. NMR Biomed.

[34] Ahn SJ, Shin HJ, Chang JH, Lee SK (2014). Differentiation between primary cerebral lymphoma and glioblastoma using the apparent diffusion coefficient: comparison of three different ROI Methods. PLoS One.

[35] Matsushima N, Maeda M, Umino M, Suzawa N, Yamada T, Takeda K (2012). Relation between FDG uptake and apparent diffusion coefficients in glioma and malignant lymphoma. Ann Nucl Med.

[36] Weller M (1999). Glucocorticoid treatment of primary CNS lymphoma. J Neurooncol.

[37] Kim EY, Kim SS (2005). Magnetic resonance findings of primary central nervous system T-cell lymphoma in immunocompetent patients. Acta Radiol.

[38] Lee HY, Kim HS, Park JW, Baek HJ, Kim SJ, Choi CG (2013). Atypical imaging features of epstein-barr virus-positive primary central nervous system lymphomas in patients without AIDS. AJNR Am J Neuroradiol.

